# Dynamic Profiling of Lipoteichoic Acid (LTA) and/or Lipopolysaccharide (LPS) Positive Extracellular Vesicles in Plasma as Diagnostic and Prognostic Biomarkers for Bacterial Infection

**DOI:** 10.1002/advs.202506613

**Published:** 2025-09-03

**Authors:** Qianqian Gao, Wenwu Zhou, Nadira Nurxat, Ke Xu, Yunyun Xu, Wei Li, Tianchi Chen, Cong Hu, Liang Dong, Qian Liu, Min Li

**Affiliations:** ^1^ Department of Laboratory Medicine, Renji Hospital, School of Medicine Shanghai Jiao Tong University Shanghai 200127 China; ^2^ Department of neurosurgery, West China Second University Hospital Sichuan University Chengdu 610044 China; ^3^ Department of cardiovascular surgery, Renji Hospital Shanghai Jiao Tong University School of Medicine Shanghai 200127 China; ^4^ Department of Urology Renji Hospital Shanghai Jiao Tong University School of Medicine Shanghai 200127 China; ^5^ School of Nursing Shanghai Jiao Tong University Shanghai 200127 China

**Keywords:** bacterial infection, biomarkers, extracellular vesicles, lipopolysaccharide, lipoteichoic acid

## Abstract

Bacterial extracellular vesicles (EVs) are emerging as promising biomarkers for diagnosing bacterial infections. This study explores the diagnostic potential of plasma EVs carrying bacterial lipopolysaccharide (LPS) or lipoteichoic acid (LTA). Using immunoelectron microscopy (IEM) and nanoflow cytometry (nano‐FCM), LTA is confirmed on cytoplasmic membrane vesicles (CMVs) from Gram‐positive bacteria and LPS on outer membrane vesicles (OMVs) from Gram‐negative bacteria, and quantified LTA‐positive or LPS‐positive (LTA^+^/LPS^+^) EVs in plasma. Bacterial infection models showed higher levels of LTA^+^/LPS^+^ EVs in infected groups compared to controls, correlating with increased inflammatory markers like interlinleukin‐6 ( IL‐6) and c‐reactive protein (CRP). Clinical samples from patients with bloodstream infections also revealed elevated LTA^+^/LPS^+^ EV levels. These EVs can distinguish bacterial infections from non‐bacterial ones, differentiate between Gram‐positive and Gram‐negative infections, and reflect disease progression and treatment response. The findings suggest that LPS^+^/LTA^+^ EVs hold strong potential as novel diagnostic and prognostic biomarkers, offering a new approach for early detection and management of bacterial infections.

## Graphical abstract

Using nano‐flow cytometry, this study analyzes the dynamic characteristics of lipoteichoic acid (LTA) and/or lipopolysaccharide (LPS) positive extracellular vesicles in the bloodstream of patients with bacterial infections, providing promising biomarkers for rapid and accurate diagnosis.

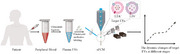

## 1 Background

Bacterial infections remain a leading cause of global morbidity and mortality, with sepsis representing the most severe clinical manifestation of bacterial invasion.^[^ ^1^ ^]^ Although significant advances have been made in sepsis diagnostics, there is currently no rapid, sensitive, and specific gold‐standard diagnostic method.^[^ ^2^, ^3^ ^]^ Bacterial culture, the conventional diagnostic approach, is characterized by its prolonged time‐to‐result, with studies indicating that up to 70.1% of bloodstream infection cases fail to identify causative pathogens promptly.^[^ ^4^, ^5^ ^]^ A retrospective analysis of 282 blood cultures from 196 ICU patients with bloodstream infections revealed that the median time to positive detection was on the 4th day postadmission.^[^ ^6^ ^]^ The advent of molecular biology techniques, particularly polymerase chain reaction (PCR) and metagenomic sequencing, has revolutionized pathogen identification by rapid and precise nucleic acid detection, thereby enhancing diagnostic sensitivity and expanding the range of pathogens.^[^ ^7^, ^8^, ^9^ ^]^

However, molecular biology techniques encounter several challenges and limitations in practical applications. Complex sample acquisition and processing workflows are the primary issue, necessitating efficient extraction and purification of bacterial nucleic acids from patient samples.^[^ ^10^, ^11^ ^]^ Furthermore, in specific circumstances such as the early stages of infection or postantibiotic treatment, detection sensitivity and specificity may be compromised due to low pathogen nucleic acid loads in the blood.^[^ ^12^ ^]^ Moreover, current techniques have limited capacity to provide a comprehensive depiction of the host's inflammatory response, which is essential for formulating personalized treatment strategies.^[^ ^13^, ^14^, ^15^ ^]^ Observational studies have shown that each hour of delay in antibiotic administration in ICU patients is associated with a 1.5% increase in mortality.^[^ ^16^ ^]^ Notably, existing monocyte volume‐based diagnostic technologies have failed to shorten sepsis diagnostic windows.^[^ ^17^ ^]^

In recent years, bacterial EVs, including Gram‐positive CMVs and Gram‐negative OMVs, have emerged as promising diagnostic biomarkers.^[^ ^18^, ^19^, ^20^ ^]^ They contain pathogen‐specific components such as LPS, LTA, proteins, nucleic acids, and metabolites, which remain stable in circulation even under antibiotic pressure.^[^ ^21^, ^22^, ^23^, ^24^, ^25^ ^]^ Microbial EVs have been detected in diverse clinical samples, including blood, urine, and cerebrospinal fluid, and their molecular cargo can differentiate Gram‐negative from Gram‐positive bacterial infections, bacterial from viral etiologies, and even identify antibiotic resistance genes.^[^ ^25^, ^26^, ^27^, ^28^ ^]^ Detection techniques such as ELISA, nanoflow cytometry, mass spectrometry, and nucleic acid assays have been applied to characterize microbial EVs in infectious diseases.^[^ ^18^, ^29^, ^30^ ^]^

Recent studies have provided compelling evidence for the diagnostic and therapeutic utility of microbial EVs.^[^ ^31^, ^32^, ^33^, ^34^ ^]^ In a murine pneumonia model, LPS⁺ OMVs labeled with polymyxin B–fluorescent probe were detectable within 6 h of infection, significantly earlier than blood culture positivity, and achieved higher diagnostic accuracy (AUC) than CRP and procalcitonin (PCT).^[^ ^35^ ^]^ In patients with sepsis, LTA⁺ EVs in plasma have demonstrated specificity for Gram‐positive bacteremia.^[^ ^34^ ^]^ Nanoflow cytometry now enables single‐particle resolution quantification of microbial EVs in clinical samples, facilitating rapid, phenotypically relevant pathogen detection.^[^ ^18^, ^36^, ^37^, ^38^, ^39^ ^]^ These advances highlight the potential of microbial EV‐based assays to shorten diagnostic windows and improve early decision‐making in sepsis management.

Despite these promising findings, the specific correlation between the dynamic changes in the number of LPS^+^/LTA^+^ EVs in plasma and the disease infection process and outcomes remains largely unexplored.

Driven by the relentless advancement and innovation in nanoflow cytometry technology, we are now empowered to precisely delineate the molecular characteristics of EVs at the single‐particle level. In this study, we focus on LPS^+^ and LTA^+^ EVs present in plasma during infection. By employing nanoflow cytometry, we investigate the dynamic alterations of LPS^+^/LTA^+^ EVs at single‐particle resolution. Our objective is to elucidate their dynamic behavior throughout the infection process and unveil their potential clinical significance in the diagnosis, prognosis, and disease monitoring of bacterial infection.

## 2 Results

### 2.1 Expression Profiles of LPS on Outer Membrane Vesicles (OMVs) and LTA on Cytoplasmic Membrane Vesicles (CMVs)

**Figure 1:**
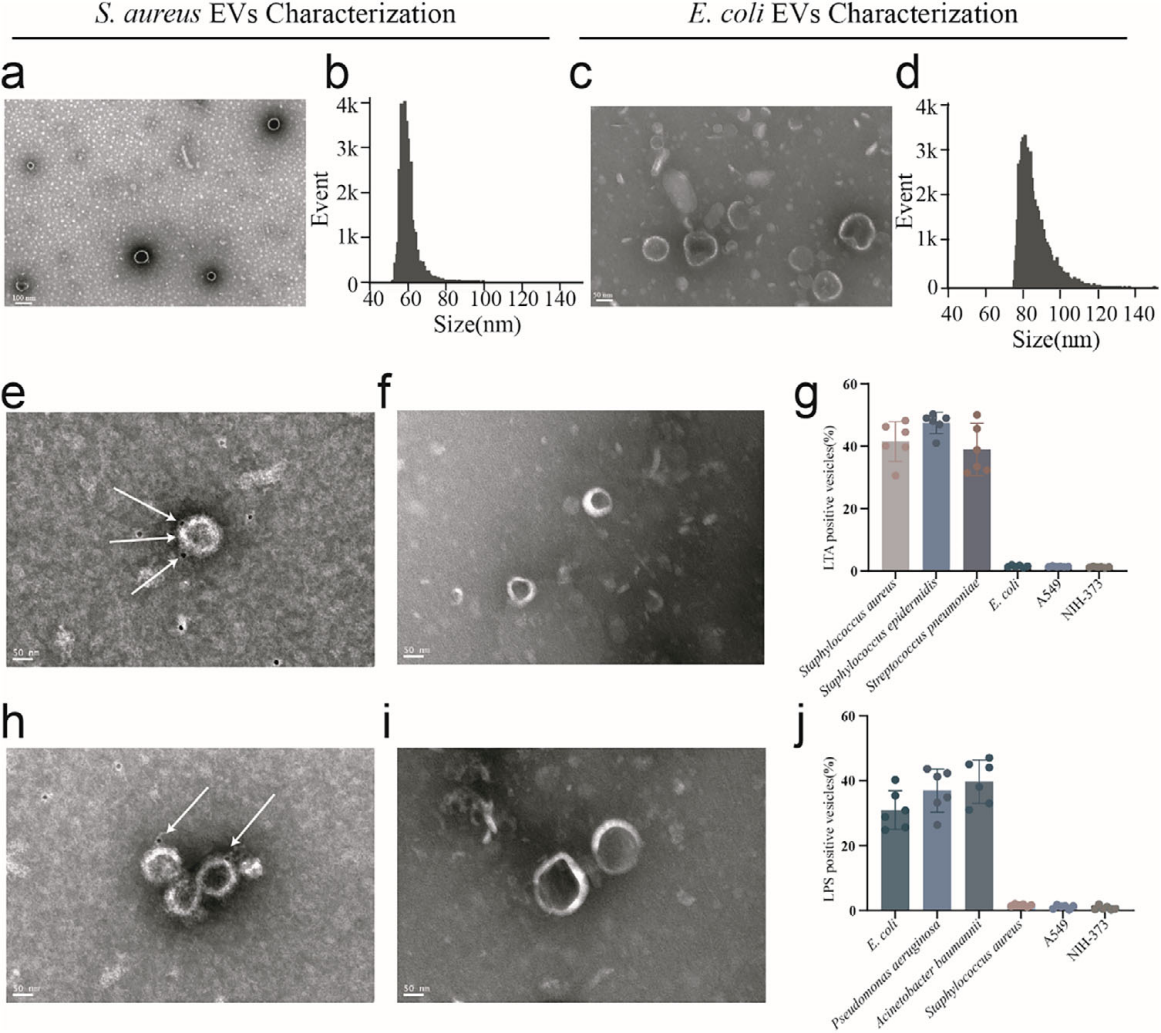
Characterization of *S. aureus‐*CMVs and *E. coli*‐OMVs a) Transmission electron microscopy (TEM) imaging of *S. aureus*‐CMVs. b) Size distribution of *S. aureus*‐CMVs determined by nano‐FCM. c) TEM imaging of *E. coli*‐OMVs. d) Size distribution of *E. coli*‐OMVs determined by nano‐FCM. e) IEM confirmation of LTA on *S. aureus*‐CMVs. CMVs were probed with anti‐LTA antibody; arrows indicate LTA^+^ CMVs. f) IEM isotype control for LTA (*S. aureus*‐CMVs + isotype IgG). g) Nano‐FCM quantification of LTA⁺ CMVs from Gram‐positive species. Data are mean ± SD, *n* = 6. h) IEM Image of LPS antibody binding to *E. coli*‐OMVs. OMVs were probed with anti‐LPS antibody; arrows indicate LPS^+^ OMVs. i) IEM isotype control for LPS (*E. coli* OMVs + isotype IgG). j) Nano‐FCM quantification of LPS⁺ OMVs from Gram‐negative species. Data are mean ± SD, *n *= 6.

Using density gradient centrifugation, we isolated OMVs from Gram‐negative *Escherichia coli* (*E. coli*) and CMVs from Gram‐positive *Staphylococcus aureus* (*S. aureus*). **Figure** **1**a illustrates the morphological structure of *S. aureus*‐CMVs, with nano‐FCM further revealing that their vesicle sizes are predominantly within the range of 50–80 nm. The morphological structure of *E. coli*‐OMVs is shown in Figure 1b, with nano‐FCM results indicating their vesicle sizes are primarily 75–120 nanometers. IEM further confirmed the specific expression of surface markers in *S. aureus*‐CMVs and *E. coli*‐OMVs. Specifically, the surface of *E. coli*‐OMVs prominently expresses LPS antigens, as shown in Figure 1c,d,f,g demonstrating that *S. aureus*‐CMVs significantly express LTA antigens.

Nano‐FCM was also employed for comprehensive analysis to quantify the expression levels of LTA on Gram‐positive CMVs and LPS on Gram‐negative OMVs. Analysis was expanded to include OMVs from Gram‐negative bacteria (*Acinetobacter baumannii, Pseudomonas aeruginosa*) and CMVs from Gram‐positive bacteria (*Staphylococcus epidermidis and Streptococcus pneumoniae*), as well as EVs from human A549 cells and mouse NIH‐3T3 cells. Nano‐FCM results show that the proportion of LPS^+^ EVs ranged from 24.8% to 40.2% (Figure 1h; Figure S1a, Supporting information) for OMVs secreted by different Gram‐negative bacteria. This proportion was significantly higher than that observed in mammalian cells (1.3%–1.7%) and Gram‐positive bacterial strains (1.6%–2.0%). Conversely, for CMVs secreted by Gram‐positive bacteria, the proportion of LTA^+^ EVs ranged from 30.6% to 48.3% (Figure 1e; Figure S1b, Supporting information). LTA was virtually undetectable in OMVs from Gram‐negative bacteria and EVs from mammalian cells, with detection rates of only 1.6%–2.2% and 0.6%–1.5%, respectively.

These findings demonstrate that LPS and LTA serve as potential markers for OMVs from Gram‐negative and CMVs from Gram‐positive bacteria, respectively, and hold great promise for future applications.

### 2.2 Interaction of LPS^+^/LTA^+^ EVs with Host Cells

To thoroughly investigate whether LPS^+^/LTA^+^ EVs can induce inflammatory responses or modulate cellular functions, the murine monocytic/macrophage cell line RAW264.7 (ATCC TIB‐71) served as the experimental model. The first step of the experiment involved extracting CMVs from *S. aureus* and OMVs from *E. coli*, yielding LTA^+^ EVs and LPS^+^ EVs, respectively. Following extraction, these LTA^+^/LPS^+^ EVs were co‐incubated with murine macrophages for 12 h under standard macrophage growth conditions (37 °C, 5% CO_2_) to allow for a comprehensive observation of their interactions.

**Figure 2:**
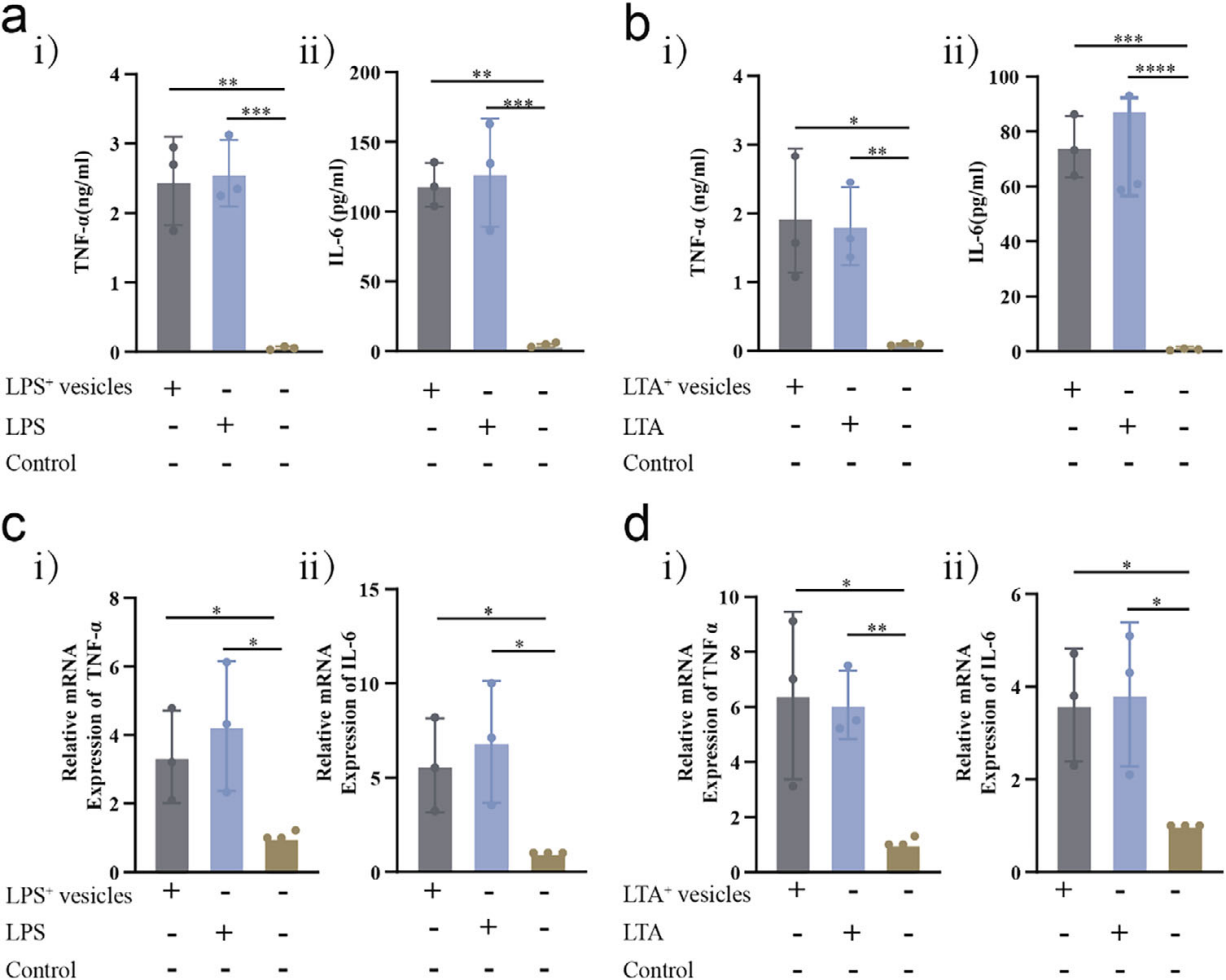
Cytokine Release and mRNA Expression Changes in Mouse Macrophages Treated with LPS^+^ EVs and LTA^+^ EVs. a) Effects of LPS^+^ EVs on TNF‐α and IL‐6 levels in mouse macrophage supernatants. i) The dynamic changes in TNF‐α levels in the supernatant of mouse macrophages treated with LPS^+^ EVs were investigated. ii) Changes in IL‐6 levels in the supernatant of mouse macrophages under the influence of LPS^+^ EVs were also observed. b) Effects of LTA^+^ EVs on TNF‐α and IL‐6 levels in mouse macrophage supernatants. i) The trends in TNF‐α levels in the supernatant of mouse macrophages treated with LTA^+^ EVs were analyzed. ii) The impact of LTA^+^ EVs on IL‐6 levels in the supernatant of mouse macrophages was assessed. c) Effects of LPS^+^ EVs on relative mRNA levels of TNF‐α and IL‐6 in mouse macrophages. i) The relative mRNA levels of TNF‐α in mouse macrophages treated with LPS^+^ EVs. ii) The relative mRNA levels of IL‐6 in mouse macrophages treated with LPS^+^ EVs. d) Effects of LTA^+^ EVs on relative mRNA levels of TNF‐α and IL‐6 in mouse macrophages. i) The relative mRNA levels of TNF‐α in mouse macrophages treated with LTA^+^ EVs. ii) The relative mRNA levels of IL‐6 mRNA in mouse macrophages treated with LTA^+^ EVs. Data are presented as mean ± SD. Statistical significance was assessed using unpaired t‐tests. Sample size (n) = 3. *p < 0.05, **p < 0.01, ***p < 0.001, ****p < 0.0001.

To quantitatively evaluate the impact of vesicles on macrophage inflammatory responses, we employed ELISA to measure the concentrations of IL‐6—a key pro‐inflammatory cytokine—and TNF‐α—a tumor necrosis factor‐α secreted by macrophages treated with LTA/LPS—in the cell culture supernatants. IL‐6 and TNF‐α are both released in significant quantities by macrophages treated with LTA/LPS. Detection and measurement allowed for a quantitative evaluation of the impact of vesicles on macrophage inflammatory responses. **Figure** **2**a,b shows a significant increase in the levels of IL‐6 and TNF‐α in the supernatants of macrophages treated with LPS^+^/LTA^+^ EVs compared to the control group, strongly suggesting that LPS^+^/LTA^+^ EVs have the ability to activate macrophages and trigger inflammatory responses.

Furthermore, we monitored expression changes of inflammation‐related genes within macrophages following treatment with LPS^+^/LTA^+^ EVs. Specifically, for both LPS^+^ EVs and LTA^+^ EVs, the expression of TNF‐α and IL‐6 genes was examined. As shown in Figure 2c,d, the experimental data clearly demonstrate the upregulated expression of these genes following vesicle treatment, further corroborating the immunostimulatory nature of the vesicles.

This study suggests that LPS^+^/LTA^+^ EVs significantly influence host immune responses through the activation of immune cells and provides compelling evidence supporting the potential role of these vesicles as inflammatory biomarkers.

### 2.3 Dynamics of LPS^+^/LTA^+^ EVs in Infected Animal Plasma

To further investigate the dynamic changes in the proportion of LTA^+^/LPS^+^ EVs in plasma during bacterial infection, we selected two clinically relevant pathogens: *S. aureus* (Gram‐positive cocci) and *E. coli* (Gram‐negative bacilli). Mouse models of pneumonia, meningitis, and bloodstream infections were established. Twelve hours post‐infection, mice's blood samples were collected and divided into two parts: one for blood culture to confirm infection status and the other for monitoring the levels of LPS^+^ EVs and LTA^+^ EVs in plasma using nano‐FCM.

Bacterial culture results showed no detectable bacteria in the blood of mice with pneumonia or meningitis (see Figure S2a–d, Supporting information), while bacteria were cultured from the bloodstream infection model (see Figure S2e,f, Supporting information). To further confirm the success of the pneumonia and meningitis models, lung and brain tissues from the respective mice were collected and stained with Hematoxylin and Eosin (H&E). Staining reveals significant inflammatory reactions at the bacterial inoculation sites in the lung and brain tissues (see Figure S3a–d, Supporting information).

**Figure 3:**
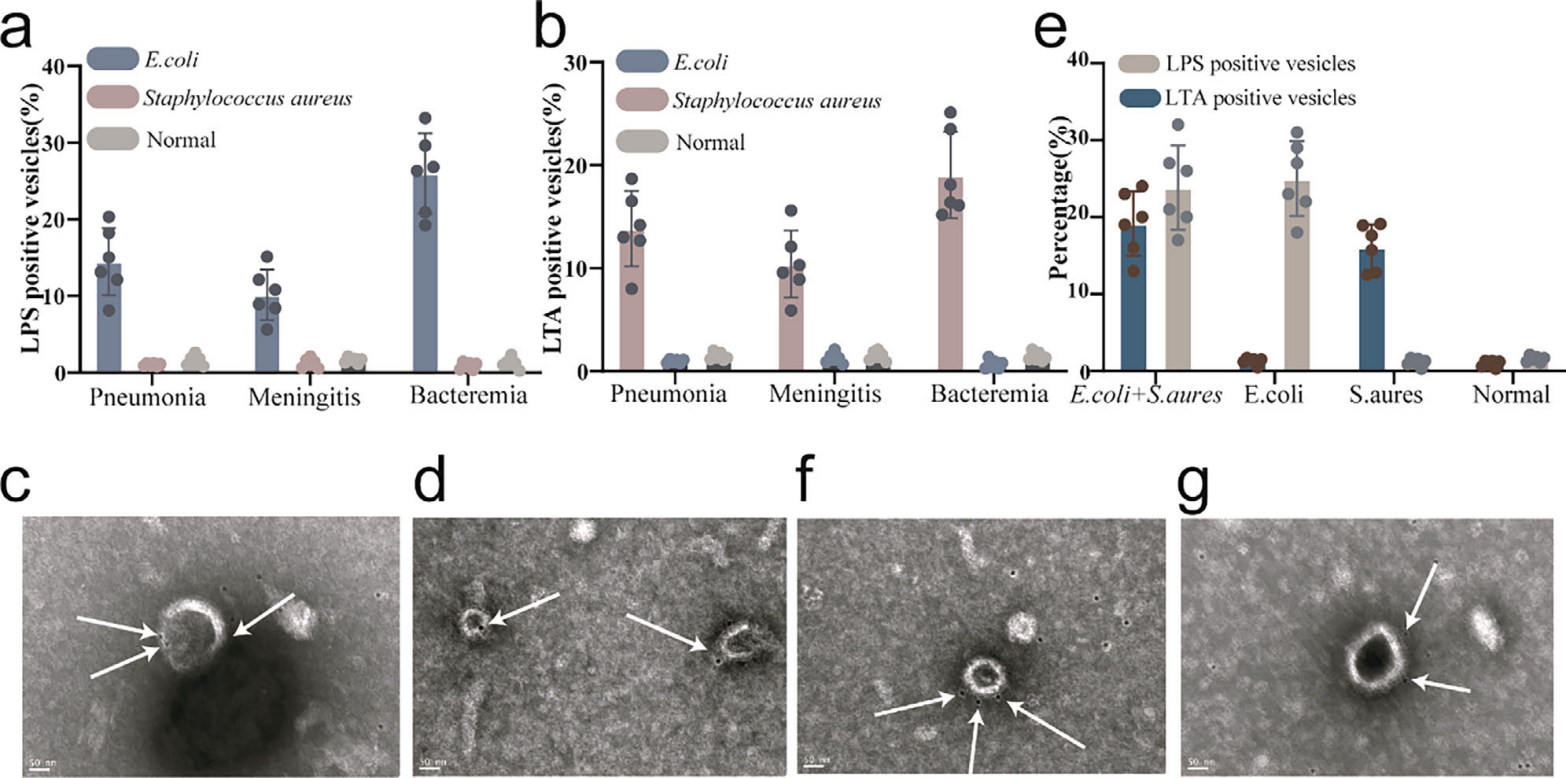
Analysis of LTA^+^/LPS^+^ EVs in Blood Post‐*S. aureus*/*E. coli* Infection in Mice a) Nano‐FCM was conducted to determine the proportion of LPS^+^ EVs in the blood of mice in various infection models induced by *E. coli*. b) Nano‐FCM was conducted to determine the proportion of LTA^+^ EVs in the blood of mice in various infection models induced by *S. aureus*. c) IEM images of LPS^+^ EVs in the blood of mice infected with *E. coli*. d) IEM images of LTA^+^ EVs in the blood of mice infected with *S. aureus*. e) Nano‐FCM analysis of the proportion of LTA^+^/LPS^+^ EVs in the blood of mice co‐infected with *S. aureus* and *E. coli*. f) IEM images of LTA^+^ EVs in the blood of mice co‐infected with *S. aureus* and *E. coli*. g) IEM images of LPS^+^ EVs in the blood of mice co‐infected with *S. aureus* and *E. coli*. The data were presented as mean values ± standard deviation. Sample size (n) = 6.

The other part of the blood samples was used to extract plasma EVs, which were then analyzed using nano‐FCM. Results show that LPS^+^/LTA^+^ EVs are detected in plasma samples from infected mice, with significantly higher levels of LTA^+^/LPS^+^ EVs in the infected group compared to the non‐infected group. The bloodstream infection group exhibits even higher levels of LTA^+^/LPS^+^ EVs than the localized infection group (see **Figure** **3**a,b).

To confirm that the LTA/LPS fluorescence signal detected by nano‐FCM originated from vesicles, the extracted vesicles were treated with 1% Triton X‐100, a nonionic surfactant. Triton X‐100 can embed into the lipid bilayer and disrupt the membrane structure, as its structure consists of a hydrophilic head and hydrophobic tail. After treatment, the proportion of LTA^+^/LPS^+^ particles decreased to ≈2% of the pre‐treatment levels (Figure S4a–f, Supporting information). This finding indicates that most LTA^+^ or LPS^+^ particles are indeed vesicle‐derived rather than lipoproteins.

IEM also reveals the presence of LPS^+^ or LTA^+^ EVs in the plasma of infected mice (see Figure 3c,d). Isotype antibody controls for IEM are shown in Figure S5a,b (Supporting information). In the mixed bloodstream infection model with Gram‐negative and Gram‐positive bacteria, we detect elevated proportions of both LPS^+^EVs and LTA^+^EVs (see Figure 3e). Bacterial culture results are shown in Figure S6 (Supporting information). IEM further shows the coexistence of LPS^+^ EVs (Figure 3f) and LTA^+^ EVs (Figure 3g) in the plasma of mice with mixed infections. Isotype antibody controls for these experiments are shown in Figure S7a,b (Supporting information).

These results indicate that during localized infections, bacteria at the site of infection can secrete vesicles into the bloodstream, and the proportion of LTA^+^/LPS^+^ EVs in plasma can serve as an indicator of bacterial infection. Bloodstream infections have a significantly elevated proportion of LTA^+^/LPS^+^ EVs in plasma than in localized infections, making this ratio a potential marker for assessing the severity of bacterial infections.

**Figure 4:**
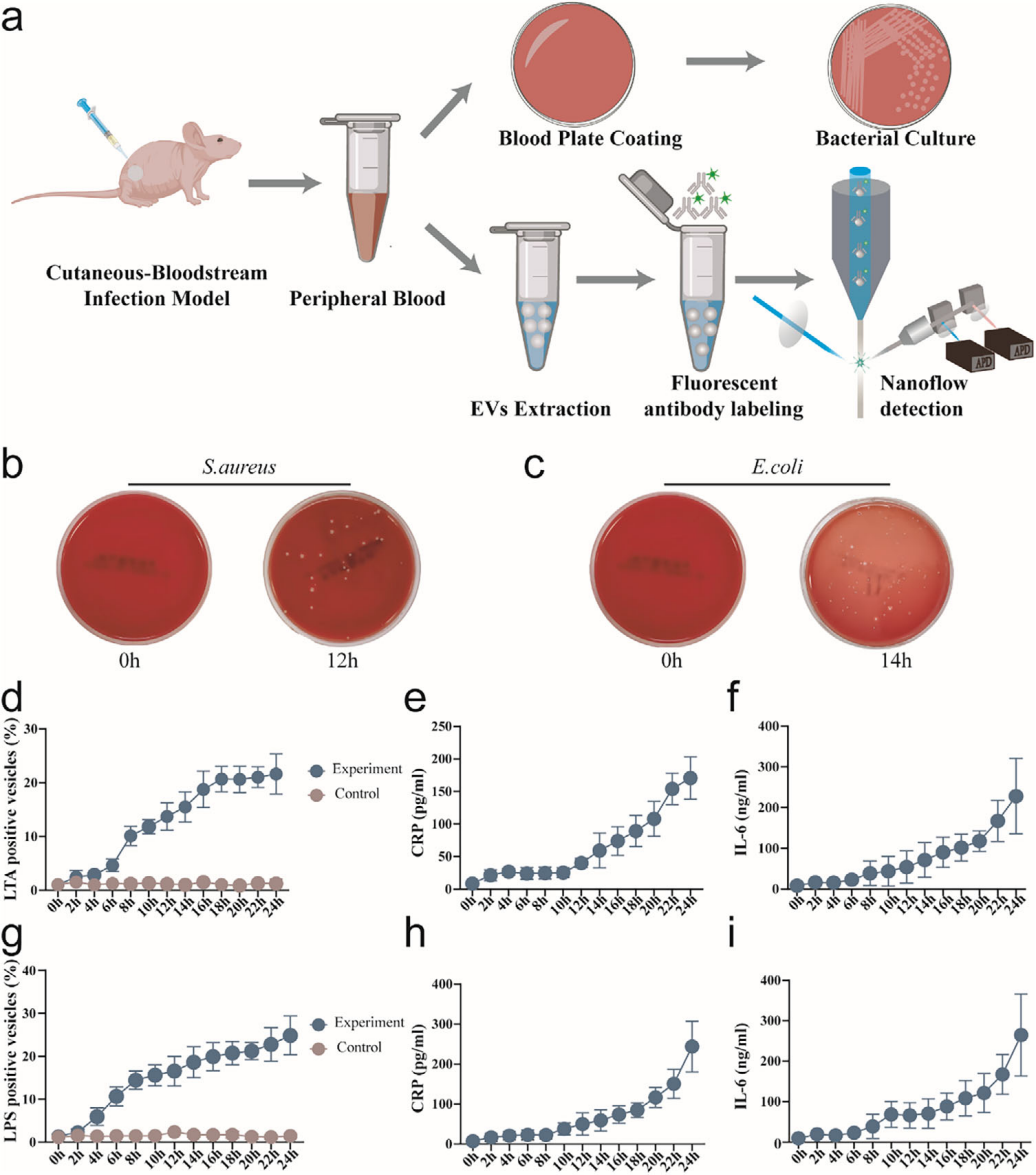
Dynamic Changes of LTA^+^/LPS^+^ EVs and Inflammatory Markers in a Skin‐Bloodstream Infection Model. a) Schematic of the skin‐bloodstream infection model in mice. This illustration depicts the construction process of the skin‐bloodstream infection model in mice. b) Blood culture results at different time points post *S. aureus* skin infection in mice. c) Blood culture results at different time points post *E. coli* infection in mice. d) Dynamic changes in the proportion of LTA^+^ EVs in mouse blood post *S. aureus* infection. e) Dynamic changes in CRP levels in blood post *S. aureus* infection. f) Dynamic changes in IL‐6 levels in blood post *S. aureus* infection. g) Dynamic changes in the proportion of LPS^+^ EVs in the blood over time post *E. coli* infection. h) Dynamic changes in CRP levels in blood post *E. coli* infection, similar to panel (e). i) Dynamic changes in IL‐6 levels in blood post *E. coli* infection, similar to panel (f). The data were presented as mean values ± standard deviation. Sample size (n) = 3. Note: The dark shadow visible behind the blood agar plate is a watermark indicating the production date and the main components of the plate.

Further investigation of the dynamic changes of LTA^+^/LPS^+^ EVs during sepsis by establishing a skin‐invasive bloodstream infection model in nude mice using *S. aureus* and *E. coli*. The experimental workflow is shown in **Figure** **4**a. Within 24 h postinfection, blood samples from the mice were collected every 2 h for blood culture and plasma vesicle extraction, and the mice were sacrificed for tissue collection. Blood culture results show that bloodstream infection developed 12 h post‐skin infection in *S. aureus*‐infected nude mice and 14 h postinfection in *E. coli*‐infected mice (Figure 4b,c).

Nano‐FCM was used to monitor the proportion of LTA^+^/LPS^+^ EVs in plasma vesicles over 24 h, while simultaneously measuring the levels of inflammatory markers IL‐6 and C‐reactive protein (CRP). The proportion of LTA^+^/LPS^+^ EVs in plasma increased with prolonged infection time, aligning with the temporal patterns of early inflammatory markers IL‐6 and CRP (Figure 4d–i). Specifically, the proportion of LTA^+^/LPS^+^ EVs began to rise 2–4 h postinfection, while inflammatory markers increased 8–10 h later. After the infection progressed to bacteremia, the proportion of LTA^+^/LPS^+^ EVs in plasma was significantly higher than in the early stages of infection.

The LTA^+^/LPS^+^ EVs precede the entry of bacteria into the bloodstream. The proportion of LTA^+^/LPS^+^ EVs may serve as an early marker of infection and reflect the intensity of inflammation. This finding offers new insights and methods for the early diagnosis and monitoring of sepsis.

### 2.4 Clinical Analysis of LTA^+^/LPS^+^ EVs in Bacterial Infection Patients

**Figure 5:**
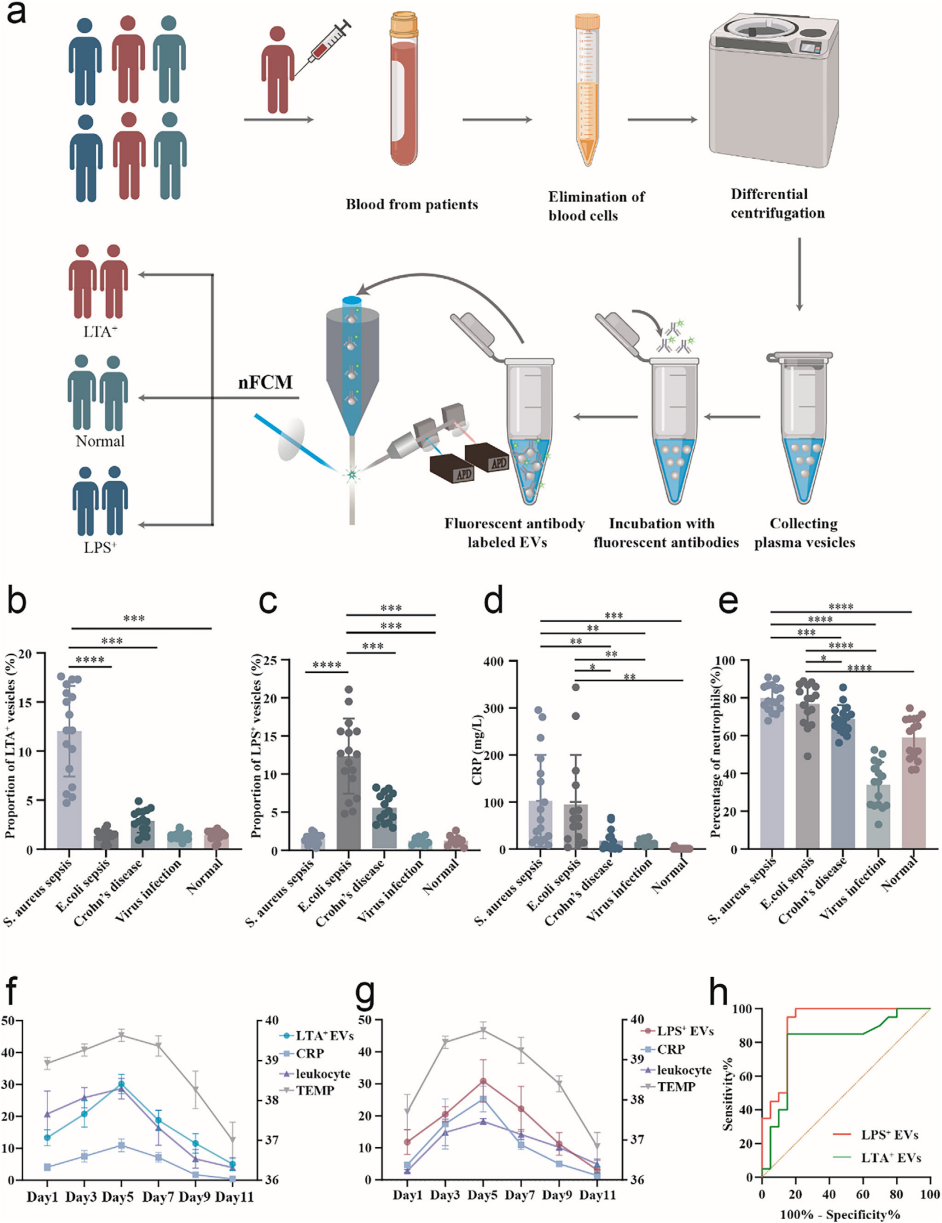
Diagnostic Value of LTA^+^/LPS^+^ EVs in Clinical Cases of *S. aureus* and *E. coli* Bacterial Infections a) Human blood sample processing workflow. b) Nano‐FCM analysis of LTA^+^ EVs proportions in plasma samples of different patients. c) Nano‐FCM analysis of LPS^+^ EVs proportions in plasma samples of different patients. d) CRP levels in plasma samples from different patients. e) Neutrophil levels in plasma samples from different patients. The statistical analysis in b), c), d), e) was performed using one‐way ANOVA. Sample size in b), c), d), e): *S. aureus* bloodstream infection (*n* = 17), *E. coli* bloodstream infection (*n* = 16), viral infection (*n* = 15), Crohn's disease (*n* = 15), healthy controls (*n* = 15). All data are presented as mean ± standard deviation. *p <0.05, **p < 0.01, ***p <0.001, ****p < 0.00 01.f) A 11‐day continuous monitoring of patients with *S. aureus* bloodstream infection, including the proportion of LTA^+^ EVs, CRP levels, white blood cell (WBC) counts, and body temperature. In the chart, body temperature data are plotted on the right vertical axis, while all other parameters are plotted on the left vertical axis. The proportion of LTA^+^ EVs is expressed as a percentage (%), CRP levels are measured in milligrams per milliliter (mg/ml), and WBC counts are given in billions per liter (10^9/L). All data are presented as mean ± standard deviation. Sample size (n) = 5. g) A 11‐day continuous monitoring of patients with *E. coli* bloodstream infection, including LPS^+^ EVs, CRP levels, WBC counts, and body temperature. The chart is constructed in the same manner as (f), with body temperature data on the right vertical axis and other parameters on the left. The units for LPS^+^ EVs proportion, CRP levels, and WBC counts are consistent with those in (f). All data are presented as mean ± standard deviation. Sample size (n) = 5. h) Receiver Operating Characteristic (ROC) curves evaluating the diagnostic performance of LTA⁺ EVs and LPS⁺ EVs in bloodstream infections.

To further explore the potential clinical value of LTA^+^/LPS^+^ EVs in the diagnosis of bacterial infections, we collected 78 clinical blood samples for in‐depth analysis. These samples encompassed a variety of disease states, including 17 patients with *S. aureus* bloodstream infections, 16 with *E. coli* bloodstream infections, 15 with viral infections, 15 with Crohn's disease, and 15 healthy controls (Supporting information, Table S1). All samples were balanced in terms of gender and age to ensure the homogeneity and comparability of the study. The processing workflow for each group of clinical samples is shown in **Figure** **5**a. Nano‐FCM reveals the following: In the *S. aureus* bloodstream infection group, the number of LTA^+^ EVs is significantly higher than in the *E. coli* bloodstream infection group, Crohn's disease group, viral infection group, and healthy controls. Conversely, in the *E. coli* bloodstream infection group, the number of LPS^+^ EVs is markedly higher than in the other groups. These results highlight the specific differences in vesicle positivity markers under different types of bacterial infections. We also observe that in both the *S. aureus* and *E. coli* bloodstream infection groups, CRP and neutrophil counts are significantly higher than in the viral infection and healthy control groups. The trends in LTA^+^/LPS^+^ EVs closely mirror the changes in these commonly used clinical inflammatory markers (see Figure 5b,c), further validating the reliability of vesicles as inflammatory biomarkers. Additionally, five patients in the *S. aureus* and *E. coli* bloodstream infection groups were clinically tracked. The results show that the proportion of LTA^+^/LPS^+^ EVs in plasma correlates with changes in clinical indicators such as body temperature, white blood cell count, and inflammatory factors (see Figure 5d,e). This finding further underscores the potential value of LTA^+^/LPS^+^ EVs as indicators for monitoring disease activity. After effective clinical antibiotic intervention, significant improvements in the relevant indicators were observed. The proportion of LTA^+^/LPS^+^ EVs in plasma dynamically changed in tandem with the improvement of the infection (see Figure 5f,g). To further explore the potential diagnostic applications of LTA⁺ EVs and LPS⁺ EVs in bloodstream infection, we constructed receiver operating characteristic (ROC) curves. The study results revealed that in the diagnosis of bloodstream infections, the area under the curve (AUC) for LTA⁺ EVs reached 0.8013, while the AUC for LPS⁺ EVs was even higher at 0.9350 (Figure 5h).

In summary, through in‐depth analysis of clinical samples, this study further validates the clinical value of LTA^+^/LPS^+^ EVs in the diagnosis of sepsis. These findings deepen our understanding of the dynamic changes of EVs during bacterial infection and provide new insights and methods for early diagnosis, disease monitoring, and prognostic assessment of bacterial infection.

## 3 Discussion

This study systematically investigated the expression characteristics of LTA in Gram‐positive CMVs and LPS in Gram‐negative OMVs and evaluated their diagnostic and prognostic potential in bacterial infections. Using nano‐FCM, we achieved high‐resolution, multiparameter analysis of individual vesicles, enabling sensitive detection of LTA⁺ and LPS⁺ EVs even in samples with low bacterial burden.

We adopted the terms “LTA⁺ EVs” and “LPS⁺ EVs” to describe plasma‐derived extracellular vesicles carrying these bacterial components. Although IEM confirms that LTA is predominantly associated with CMVs from Gram‐positive bacteria and LPS with OMVs from Gram‐negative bacteria, plasma‐derived EVs may originate from multiple sources, including bacterial secretion, bacterial lysis, adsorption of bacterial components onto host EVs, or processing by immune cells. Given this heterogeneity and the inability to directly trace their origin without visualization, a marker‐based nomenclature more accurately reflects our detection approach and avoids overinterpretation of their precise biogenesis. The use of microbial EVs as disease biomarkers is not unprecedented. Several studies have explored microbial EV‐based diagnostic approaches in various clinical contexts.^[^ ^40^, ^41^, ^42^ ^]^ These pioneering works established the foundation for the diagnostic application of microbial EVs. Building on this groundwork, our study advances the field by i) directly comparing LTA^+^ and LPS^+^ EVs in both Gram‐positive and Gram‐negative bacterial infections, ii) analyzing their dynamics in ulcerative colitis, bacterial infections, and nonbacterial gastrointestinal diseases, and iii) leveraging nano‐FCM for rapid (within 3 h) and highly sensitive single‐vesicle profiling. This combination of bacterial specificity, speed, and multiparameter analysis represents an extension beyond prior work.

We acknowledge that UC‐EVs likely comprise a heterogeneous mixture of vesicles from host cells, bacteria, and possibly fungi.^[^ ^43^, ^44^ ^]^ Such complexity reflects the diverse origins of EVs in biofluids and may affect biomarker specificity and quantification. LTA and LPS are unique to bacteria^[^ ^45^, ^46^ ^]^ and absent in mammalian cells, which supports their use as specific diagnostic targets; in our cohort, patients with non‐bacterial gastrointestinal diseases exhibited minimal to undetectable LTA^+^ and LPS^+^ EV levels. The specificity of these markers was further supported by Triton X‐100 disruption assays, in which >95% of LTA^+^/LPS^+^ particle signals were lost after membrane disruption, confirming their vesicular origin. Nevertheless, potential cross‐reactivity from nontarget microbial EVs or structurally similar host components cannot be fully excluded.^[^ ^47^ ^]^

The sensitivity of LTA^+^ and LPS^+^ EV detection can be influenced by several factors, such as inappropriate storage, repeated freeze–thaw cycles, or prolonged storage at non‐optimal temperatures may degrade EVs.^[^ ^48^, ^49^ ^]^ Different bacterial species release EVs at varying rates and with different LTA/LPS content; rare species with low marker levels may escape detection.^[^ ^50^ ^]^ Even under these constraints, the nano‐FCM platform detected LTA^+^ and LPS^+^ EVs in diluted clinical samples, demonstrating robust analytical sensitivity. Importantly, LPS^+^ EVs achieved high diagnostic accuracy for Gram‐negative bacteremia (AUC = 0.9350; sensitivity = 90%–95%; specificity = 85%–90%), whereas LTA^+^ EVs showed moderate but complementary performance for Gram‐positive infections (AUC = 0.8013; sensitivity = 70%–80%; specificity = 70% –85%). Compared with conventional blood culture,^[^ ^51^ ^]^ which has high specificity but limited sensitivity (≈60%–80%) and slow turnaround (>24 h), and molecular assays such as PCR,^[^ ^52^ ^]^ which can achieve sensitivities of 80%–95% but require prior knowledge of target pathogens, the Nano‐FCM approach enables rapid, broad‐range detection of microbial EVs without pathogen pre‐identification. However, cross‐reactivity from non‐target microbial EVs or host‐derived vesicles cannot be completely excluded.^[^ ^47^ ^]^ Additional purification strategies, such as immunoaffinity isolation or density gradient centrifugation, may further enhance specificity.

Isolating pure microbial EVs remains a technical challenge. While UC is widely used, it co‐isolates heterogeneous EV populations.^[^ ^53^ ^]^ Additional enrichment methods—such as immunoaffinity capture^[^ ^54^ ^]^ or density gradient ultracentrifugation^[^ ^55^ ^]^—may help improve purity and specificity, particularly when targeting LTA⁺ or LPS⁺ vesicles. Future strategies may combine methods, e.g., SEC for bulk impurity removal followed by antibody‐based capture of LTA⁺ or LPS⁺ EVs, to achieve both high purity and specificity. Integration with nano‐FCM could enable direct, rapid, and multiparameter analysis of enriched microbial EV populations.

This study is limited by a relatively small sample size, which may affect generalizability. Larger multicenter cohorts are needed to validate the robustness of our findings. Mechanistic studies should further clarify the release pathways of LTA⁺ and LPS⁺ EVs and their interactions with host immune cells, potentially revealing novel therapeutic targets. Our findings underscore the potential of LTA⁺ and LPS⁺ EVs as rapid, specific, and sensitive biomarkers for bacterial infections, particularly in differentiating Gram‐negative from Gram‐positive bacteremia. By acknowledging existing literature, addressing UC‐EV complexity, evaluating sensitivity and specificity, and outlining purification strategies, this work provides both practical diagnostic insights and a roadmap for future research aimed at clinical translation.

## 4 Experimental Section

### Bacterial Cultures

Bacterial strains used in this study consisted of both Gram‐positive and Gram‐negative bacteria. Gram‐positive bacteria included *S. aureus*, *Staphylococcus epidermidis*, and *Streptococcus pneumoniae*, while the Gram‐negative bacteria included *E. coli*, *Pseudomonas aeruginosa*, and *Acinetobacter baumannii*.

For the cultivation of Gram‐positive bacteria, Tryptic Soy Broth (TSB) was employed as the culture medium. The bacteria were cultured at 37 °C with shaking at 200 rpm to ensure uniform growth under optimal temperature and aeration conditions.

For Gram‐negative bacteria, Luria‐Bertani (LB) was selected as the culture medium. The cultivation was also conducted at 37 °C with shaking at 200 rpm to facilitate rapid bacterial proliferation and growth.

### Density Gradient Centrifugation for Isolation of S. aureus‐CMVs and E. coli‐OMVs

*S. aureus* and *E. coli* were cultured overnight in TSB/LB at 37 °C with shaking at 200 rpm. The overnight cultures were diluted 1:100 into fresh TSB/LB and incubated under the same conditions for 8 h to reach the logarithmic growth phase and promote *S. aureus*‐CMV and *E. coli*‐OMV secretion. Bacterial cultures were centrifuged at 4000 × g for 30 min at 4 °C (Beckman Coulter Allegra X‐15R, USA) to pellet bacterial cells. The resulting supernatant was passed through a 0.22 µm pore‐size filter membrane (Millipore, #SLGPR33RB, USA) to remove residual bacteria. The clarified supernatant was concentrated using an Amicon Ultra centrifugal filter unit (100 kDa molecular weight cutoff; Millipore, UFC9100, USA) to reduce volume and remove small molecules and free proteins. The concentrated sample was first ultracentrifuged at 174 900 × g for 4 h at 4 °C using an SW 32Ti rotor (Beckman Coulter Optima XE‐90, USA) to pellet total vesicles. The pellet was resuspended in 50% OptiPrep (iodixanol) solution (Sigma, #D1556, Germany), overlaid sequentially with 40% and 10% OptiPrep solutions to establish a discontinuous density gradient, and ultracentrifuged at 200 000 × g for 16 h at 4 °C using an SW 41Ti rotor (Beckman Coulter). The visible band at the interface between the 10% and 40% OptiPrep layers was carefully aspirated, transferred to a clean tube, and stored at −80 °C for downstream experiments.

### Isolation of Plasma EVs by Differential Centrifugation

Peripheral venous blood (2 mL from patients;500 µL from mice) from each was collected into EDTA‐coated tubes and processed within 2 h. Plasma was obtained by centrifugation at 1000 × g for 10 min at 4 °C, followed by two spins at 2500 × g for 15 min to remove residual cells, platelets, and debris. The supernatant was centrifuged at 10 000 × g for 20 min to deplete apoptotic bodies and large vesicles, filtered through a 0.45 µm membrane (Sigma, Cat#SLHPR33RB, Germany), and ultracentrifuged at 100 000 × g for 2 h at 4 °C (Beckman Coulter Optima XE‐90, SW55Ti rotor, USA). Pellets were washed once in phosphate‐buffered saline (PBS; Sangon Biotech, Shanghai, China) under the same conditions, resuspended in 100 µL PBS, aliquoted, and stored at −80 °C.

### Nano‐Flow Cytometry Detection of LPS^+^ and LTA^+^ Extracellular vesicles

In this study, we refer to vesicles carrying LTA or LPS as “LTA⁺ EVs” and “LPS⁺ EVs” rather than “LTA⁺ CMVs” or “LPS⁺ OMVs,” since plasma‐derived EVs originate from heterogeneous sources and cannot be definitively assigned to a specific bacterial subtype without direct visualization.

EVs were immuno‐stained and analyzed under standardized conditions using a Nano‐Flow Cytometry system (Nano‐FCM Inc., Model N30E). Prior to antibody incubation, all samples were diluted to 5 × 10⁸ particles /mL in PBS (100 µL reaction volume, ≈5 × 10⁷ particles per assay) to ensure identical antigen‐to‐antibody ratios across experiments. Experimental aliquots were incubated with either a primary rabbit polyclonal anti‐LTA antibody (Life Span Biosciences, LS‐C760114) or a rabbit polyclonal anti‐LPS antibody (Life Span Biosciences, LS‐C343196) and incubated at 37 °C for 60 min. Excess primary antibody was removed by two consecutive rounds of ultracentrifugation at 120 000 × g for 60 min at 4 °C (SW55Ti rotor, Beckman Coulter XE‐90K). Pellets were gently resuspended in 100 µL PBS and subsequently incubated with Alexa Fluor 488‐conjugated goat anti‐rabbit IgG (Abcam, ab150113) for 1 h at 37 °C. Unbound secondary antibody was removed by two additional ultracentrifugation steps under identical conditions, and the final pellet was resuspended in 20 µL PBS. Negative controls were processed in parallel and included: 1) EVs incubated with secondary antibody only; 2) EVs incubated with primary antibody only. 3) EVs incubated with isotype‐matched IgG (Life Span Biosciences, LS‐C292295) followed by secondary antibody.

Nano‐FCM measurements were performed with exactly 10 000 individual EV particles per sample. The sheath fluid flow rate was set to 40 pL min^−1^ and the sample flow rate to 2 nL min^−1^, yielding a 25 fL detection volume. A calibration curve generated using 100 nm non‐antibody‐coated fluorescent polystyrene microspheres was applied to convert absolute particle counts into percentages of positive events. All samples were analyzed under identical instrument settings to ensure cross‐sample comparability.

### Transmission Electron Microscopy (TEM)

A 10 µL aliquot of EV suspension was placed onto a carbon‐coated 200‐mesh copper grid (Zhongjingkeyi, #BZ110125b, Beijing, China) and allowed to adsorb for 1 min at room temperature (RT). Excess liquid was removed with filter paper, and the grid was fixed with 0.4 µL of 25% glutaraldehyde for 60 s. Samples were then negatively stained with 2% uranyl acetate for 90 s, air‐dried, and examined using a Tecnai G2 Spirit BioTWIN transmission electron microscope (Thermo Fisher Scientific, USA). Images were acquired with a Megaview G2 CCD camera and processed using Adobe Photoshop.

### Immunoelectron Microscopy (IEM)

EVs were mixed 1:1 (v/v) with 4% paraformaldehyde, and 10–20 µL of the mixture was placed onto Parafilm. A carbon‐coated grid was floated (film‐side down) on the droplet for 20 min to capture EVs, followed by the following sequential steps: 1) Washing: Two 3‐min washes in 100 µl PBS; 2) Aldehyde quenching: three incubations in 50 mM glycine (3 min each); 3) Blocking: Overnight blocking at 4 °C with 5% BSA; 4) Primary antibody incubation: 1 h at RT with mouse anti‐LPS (Novus, Cat#NB100‐62450) or anti‐LTA (Novus, Cat#NBP1‐60146) diluted 1:20 in blocking buffer; 5) Washing: Six washed with PBS (3 min each); 6) Secondary antibody incubation: 1 h at RT with colloidal‐gold–conjugated secondary antibody (Abcam, Cat#ab29619; 1:200). 7) Washing: Six washes with 0.5% BSA (3 min each), followed by six PBS washes (2 min each); 8) Post‐fixation: 2 min in 1% glutaraldehyde; 9) Final rinsing: Six rinses in double‐distilled water (2 min each). Grids were counterstained with 2% uranyl acetate for 90 s, air‐dried, and imaged as described for TEM.

### EV‐Induced Cytokine Release Assay (ELISA)

RAW264.7 macrophages (2×10⁵/well, 24‐well plate) were serum‐starved overnight, then exposed to 10 µg mL^−1^ purified EVs (≈1 × 10⁹ particles/well) or an equal volume of PBS (negative control) for 12 h at 37 °C in a humidified atmosphere containing 5% CO_2_. Following treatment, culture supernatants were collected by centrifugation at 300 × g for 5 min at 4 °C to remove cell debris. Concentrations of IL‐6 and TNF‐α were quantified using commercially available mouse ELISA kits (R&D Systems; IL‐6: M600B, TNF‐α: MTA00B) according to the manufacturer's instructions.

### Gene‐Expression Analysis by Quantitative PCR (qPCR)

Total RNA was isolated from cells using TRIzol–chloroform method, followed by precipitation with isopropanol, washing with 75% ethanol, and resuspension in DEPC‐treated water. RNA concentration and purity (A_260_/A_280_ 1.9–2.1) were assessed using a NanoDrop spectrophotometer, and only samples with an A_260_/A_280_ ratio between 1.9 and 2.1 were used for subsequent analysis. RNA samples were either reverse‐transcribed immediately or stored at −80 °C until use.

For cDNA synthesis, 1 µg of total RNA was reverse‐transcribed using HiScript II RT SuperMix kit (Vazyme, R222‐01; Vazyme Biotech Co., Ltd., Nanjing, China) according to the manufacturer's instructions. Quantitative PCR was performed in a 20 µL reaction volume containing SYBR Green Master Mix (Vazyme, Q711‐02) on a Roche LightCycler 96 system under the following cycling conditions: initial denaturation at 95 °C for 30 s; 40 cycles of 95 °C for 5 s and 60 °C for 30 s; followed by a melting curve analysis from 65 to 95 °C. Primers were as follows: IL‐6: F 5′‐ GAG GAT ACC ACT CCC AAC AGA CC‐3′, R5’‐ AAG TGC ATC ATC GTT GTT CAT ACA‐3′; TNF‐α:F 5′‐TCC TTC TCA TTC CTG CTT GTG G‐3′ R5’‐GGT CTG GGC CAT AGA ACT GA‐3′; β‐actin: F 5′‐GGA GGG GGT TGA AGG TCT TTA‐3′,R 5′‐AGG TGT AAA ACG CAG CTC AGT A‐3′. Relative expression was normalized to β‐actin and calculated by the 2^−ΔΔCt method; All qPCR reactions were performed in triplicate.

### Pneumonia Model in BALB/c Mice

Bacterial suspensions of *S. aureus* or *E. coli* (4×10^8 CFU mL^−1^) were prepared in sterile PBS. Mice were gently restrained, and 20 µL of suspension was instilled into the left nostril using a pipette. Following instillation, mice were kept upright for ≈1 min to promote distribution.

Mice were then divided into nine time‐point groups post‐infection: 0 h (healthy control), 6 h, 12h, 18h, 24h, 30h, 36h, 48h and 72 h (*n* = 6 per group). At each time point, 1 mL of blood was collected from the retro‐orbital plexus into EDTA‐coated tubes. Mice were then euthanized by CO_2_ inhalation.

Half of each blood sample (500 µL) was plated onto blood agar plates and incubated at 37 °C for 24 h to assess bacterial growth by culture. The remaining 500 µL was processed for extracellular vesicle (EV) isolation from plasma using differential centrifugation. Isolated EVs were stored at −80 °C until further analysis.

### Meningitis Model in BALB/c Mice

Mice were anesthetized until complete loss of reflexes and positioned on a stereotaxic surgical platform. A precise skin incision was made 1–2 mm to the back from the bregma point, and ≈1 mm lateral to the midline. Ophthalmic scissors were used to drill a small hole through the skull to avoid any damage to the brain tissue. With the aid of a needle holder, the needle was gently inserted through the hole and accurately advanced into the lateral ventricle of the brain.

Mice were randomly assigned to experimental groups (*n* = 6 per group). 2 µL of bacterial suspension containing 1×10^9 CFU of *S. aureus* or *E. coli*, was slowly injected into the lateral ventricle of the brain. Control animals received an equal volume of sterile PBS. After the injection, the needle was withdrawn carefully to minimize cerebrospinal fluid leakage and avoid air bubbles within the cranial cavity.

Following the injection, the cranial hole and skin incision were meticulously sutured to close the surgical site. Antibiotic ointment was applied to the wound to prevent postoperative infection.

At 24 h post‐injection, 1 mL of blood was collected via retro‐orbital puncture, and mice were euthanized for brain tissue collection. The blood samples were divided into two equal portions: 500 µL was cultured to confirm the absence of bacteremia, and the remaining 500 µL was processed for extracellular vesicle (EV) isolation by differential centrifugation. Purified EVs were stored at −80 °C for subsequent analyses. Brain tissues were fixed and processed for H&E staining to evaluate bacterial infection and tissue pathology.

### Bacteremia Model in BALB/c Mice

A sepsis model in BALB/C mice was created via retro‐orbital injection. Under anesthesia, bacteria were injected (either *S. aureus* ATCC25923 or *E. coli* ATCC25923) at a concentration of 10^8 CFU mL^−1^ into the retro‐orbital sinus of the mice at a 45° angle. Each experimental group consisted of six mice, with an equal number of healthy mice serving as controls.

At 24 h post‐injection, 1 mL of blood was collected from each mouse via retro‐orbital puncture and transferred immediately into EDTA‐coated tubes. Mice were then euthanized. Of the collected blood, 500 µL was used for bacterial culture to assess the success of the sepsis model, while the remaining 500 µL was utilized for the isolation of EVs from the mouse plasma. The isolated EVs were stored at −80 °C for subsequent analyses.

### Skin‐Invasive Bloodstream Infection Model in BALB/c Mice

A skin abscess model was established in BALB/c nude mice via subcutaneous injection. After anesthetizing the mice, the skin at the injection site was gently lifted, and a sterile needle was inserted upward into the subcutaneous tissue. The injection was performed swiftly to prevent leakage of the bacterial suspension. A standardized dose of 200 µL suspension containing 2×10^8 CFU mL^−1^ of *S. aureus* or *E. coli* was administered for this experiment. Control mice received an equal volume of PBS.

The mice were then randomly divided into 13 groups (*n* = 6 per group). From each group, 1 mL of blood was collected at designated 2‐h intervals via retro‐orbital puncture, after which the animals were euthanized. Of the collected blood, 500 µL was used for bacterial culture to monitor the infection status, while the remaining 500 µL was used to measure and record the levels of inflammatory factors in the plasma. Differential centrifugation techniques to isolate EVs from the plasma were used. The isolated EVs were aliquoted and stored at −80 °C for subsequent analysis.

### Ethics Statement

The animal study has undergone rigorous review by the Ethics Committee of Renji Hospital, Shanghai Jiao Tong University School of Medicine in Shanghai, China, and has been approved (Ethics Number: IACUC‐2022‐Mi‐205). Collection of human plasma samples during this study strictly adhered to ethical standards and relevant laws and regulations. Our study was approved by the Shanghai Jiaotong University School of Medicine, Renji Hospital Ethics Committee, with the approval number RA‐2024‐306. All samples were derived from residual blood remaining after routine medical testing, ensuring no interference with the original medical use and purpose of the samples. According to the guidelines established by the ethics review committee, the scientific use of such discarded, unidentifiable residual blood samples that do not contain any personal identification information has been explicitly exempted, and no separate informed consent is required.

## Conflict of Interest

The authors declare no conflict of interest.

## Author Contributions

Q.Q. and W.W. conceived and designed the study, conducted the majority of the experiments, and prepared the initial manuscript. N.D.L., K. X., and Y.Y. were instrumental in organizing the clinical data, ensuring the study's smooth progression. Q.Q. and W.W. collectively optimized the experimental protocols and analyzed the data. W.L., X.J., Y.F., C.H., L.D., and Q.L. contributed to the writing of the manuscript. M.L. participated in the development of the concepts and methods, secured the necessary resources, and played key roles in writing and revising the manuscript. M.L. also oversaw project management and provided financial support to ensure the study's completion. All authors reviewed and approved the final version of the manuscript. Q.G. and W.Z. contributed equally to this work.

## Supporting information



Supporting Information

## Data Availability

The data that support the findings of this study are available from the corresponding author upon reasonable request.

## References

[1] I. P. C. Group , Lancet Infect. Dis. 2024, 24, 868.38640940

[2] D. M. Hill , M. D. Percy , S. R. Velamuri , J. Lanfranco , I. Romero Legro , S. E. Sinclair , W. L. Hickerson , J. Burn Care Re. 2018, 39, 982. 10.1093/jbcr/iry022 29771353

[3] M. Shankar‐Hari , G. S. Phillips , M. L. Levy , C. W. Seymour , V. X. Liu , C. S. Deutschman , D. C. Angus , G. D. Rubenfeld , M. Singer , JAMA, J. Am. Med. Assoc. 2016, 315, 775. 10.1001/jama.2016.0289 PMC4910392 26903336

[4] D. C. Vidya Iyer , B. Bipin Malla , A. R. Panda , G. Rabson , N. Horowitz , K. Heger , A. Gupta , E. R. N. Singer , J. Clin. Microbiol. 2024, 62, 0149823. 10.1128/jcm.01498-23 PMC10935643 38315022

[5] C. J. Paoli , M. A. Reynolds , M. Sinha , M. Gitlin , E. Crouser , Crit. Care Med. 2018, 46, 1889. 30048332 10.1097/CCM.0000000000003342 PMC6250243

[6] B. K. Hogan , S. E. Wolf , D. R. Hospenthal , L. C. D'Avignon , K. K. Chung , H. C. Yun , E. A. Mann , C. K. Murray , J. Burn Care Res. 2012, 33, 371. 22210056 10.1097/BCR.0b013e3182331e87

[7] M. Berry , I. Melendrez , M. C. , K. A. Bishop‐Lilly , W. Rutvisuttinunt , S. Pollett , E. Talundzic , L. Morton , R. G. Jarman , J. Infect. Dis. 2020, 221, 292. 10.1093/infdis/jiz286 31612214

[8] P. Sinha , V. E. Kerchberger , A. Willmore , J. Chambers , H. Zhuo , J. Abbott , C. Jones , N. Wickersham , N. Wu , L. Neyton , C. R. Langelier , E. Mick , J. He , A. Jauregui , M. M. Churpek , A. D. Gomez , C. M. Hendrickson , K. N. Kangelaris , A. Sarma , A. Leligdowicz , K. L. Delucchi , K. D. Liu , J. A. Russell , M. A. Matthay , K. R. Walley , L. B. Ware , C. S. Calfee , Lancet Respir. Med. 2023, 11, 965. 37633303 10.1016/S2213-2600(23)00237-0 PMC10841178

[9] V. L. Baillie , S. A. Madhi , V. Ahyong , C. P. Olwagen , Nat. Commun. 2023, 14, 5373. 37666833 10.1038/s41467-023-40958-8 PMC10477270

[10] S. Brodzka , P. Kaminski , J. Baszynski , S. Mroczkowski , K. Rektor , E. Stanek , J. Kwiecinska‐Pirog , R. Grochowalska , N. Kurhaluk , H. Tkaczenko , Cell. Physiol. Biochem. 2025, 59, 47. 40079913 10.33594/000000756

[11] D. G. Maghini , E. L. Moss , S. E. Vance , A. S. Bhatt , Nat. Protocal. 2021, 16, 458. 10.1038/s41596-020-00424-x PMC8750633 33277629

[12] M. van den Brand , F. A. M. van den Dungen , M. P. Bos , M. M. van Weissenbruch , A. M. van Furth , A. de Lange , A. Rubenjan , R. P. H. Peters , P. H. M. Savelkoul , Crit. Care 2018, 22, 105. 29679983 10.1186/s13054-018-2010-4 PMC5911371

[13] E. J. Giamarellos‐Bourboulis , A. Siampanos , A. Bolanou , S. Doulou , N. Kakavoulis , K. Tsiakos , S. Katopodis , G. Schinas , L. Skorda , Z. Alexiou , K. Armenis , P. Katsaounou , G. Chrysos , A. Masgala , G. Poulakou , N. Antonakos , A. Safarika , M. Kyprianou , K. Dakou , S. Gerakari , I. C. Papanikolaou , H. Milionis , M. Marangos , G. N. Dalekos , V. Tzavara , K. Akinosoglou , E. Hatziaggelaki , S. Sympardi , T. Kontopoulou , M. Mouktaroudi , et al., Lancet Respir. Med. 2024, 12, 294. 38184008 10.1016/S2213-2600(23)00412-5

[14] A. J. Kwok , A. Allcock , R. C. Ferreira , E. Cano‐Gamez , M. Smee , K. L. Burnham , Y.‐X. Zurke , A. Novak , M. Darwent , T. Baron , C. Brown , S. Beer , A. Espinosa , T. Panduro , D. Georgiou , J. Martinez , H. Thraves , E. Perez , R. Fernandez , A. Sobrino , V. Sanchez , R. Magallano , K. Dineen , J. Wilson , S. McKechnie , A. J. Mentzer , C. Monaco , I. A. Udalova , C. J. Hinds , J. A. Todd , et al., Nat. Immunol. 2023, 24, 767. 37095375 10.1038/s41590-023-01490-5

[15] C. Tan , H. Ma , J. Chen , G. Ma , A. Jha , S. Tan , Y. Zhu , M. Liu , K. Liu , X. Xiao , M. Aziz , H. Chen , P. Wang , H. Zhang , Adv. Sci. 2025, 02297. 10.1002/advs.202502297 PMC12533375 40704655

[16] L. T. Cardoso , C. M. Grion , T. Matsuo , E. H. Anami , I. A. Kauss , L. Seko , A. M. Bonametti , Crit. Care 2011, 15, 28. 10.1186/cc9975 PMC3222064 21244671

[17] Y. H. Huang , C. J. Chen , S. C. Shao , C. H. Li , C. H. Hsiao , K. Y. Niu , C. C. Yen , Crit. Care Med. 2023,51, 106. 10.1097/CCM.0000000000005820 PMC10090344 36877030

[18] Q. Gao , W. Zhou , Z. Shen , T. Chen , C. Hu , L. Dong , D. Han , M. Li , J. Extracell. Vesicles 2025, 14, 70111. 10.1002/jev2.70111 PMC12269531 40673893

[19] M. Sun , J. Yang , Y. Fan , Y. Zhang , J. Sun , M. Hu , K. Sun , J. Zhang , Adv. Sci. 2023, 10, 2303617. 10.1002/advs.202303617 PMC10646251 37749882

[20] R. P. Carney , R. R. Mizenko , B. T. Bozkurt , N. Lowe , T. Henson , A. Arizzi , A. Wang , C. Tan , S. C. George , Nat. Nanotechnol. 2025, 20, 14. 39468355 10.1038/s41565-024-01774-3 PMC11781840

[21] R. Kalluri , V. S. LeBleu , Science 2020, 367, 6977. 10.1126/science.aau6977 PMC7717626 32029601

[22] V. Weinberger , B. Darnhofer , H. B. Thapa , P. Mertelj , R. Stentz , E. Jones , G. Grabmann , R. Mohammadzadeh , T. Shinde , C. Karner , J. Ober , R. Juodeikis , D. Pernitsch , K. Hingerl , T. Zurabishvili , C. Kumpitsch , T. Kuehnast , B. Rinner , H. Strohmaier , D. Kolb , K. Gotts , T. Weichhart , T. Köcher , H. Köfeler , S. R. Carding , S. Schild , Nat. Commun. 2025, 16, 5094. 40461460 10.1038/s41467-025-60271-w PMC12134236

[23] Y. Xue , C. Yu , H. Ouyang , J. Huang , X. Kang , J. Am. Chem. Soc. 2024, 146, 11906. 38629727 10.1021/jacs.4c00889

[24] N. J. Bitto , L. Cheng , E. L. Johnston , R. Pathirana , T. K. Phan , I. K. H. Poon , N. M. O'Brien‐Simpson , A. F. Hill , T. P. Stinear , M. Kaparakis‐Liaskos , J. Extracell. Vesicles 2021, 10, 12080. 10.1002/jev2.12080 PMC8015888 33815695

[25] F. Andreoni , M. Toyofuku , C. Menzi , R. Kalawong , S. Mairpady Shambat , P. François , A. S. Zinkernagel , L. Eberl , Antimicrob. Agents Chemother. 2019, 63, 01439. 10.1128/AAC.01439-18 PMC6355553 30509943

[26] Y. Choi , J. P. , A. Jo , C. W. Lim , J. M. Park , J. W. Hwang , K. S. Lee , Y. S. Kim , H. Lee , J. Moon , Sci. Adv. 2025, 11, 11. 10.1126/sciadv.ado6894 PMC11691634 39742488

[27] F. Bu , X. Shen , H. Zhan , D. Wang , L. Min , Y. Song , S. Wang , J. Am. Chem. Soc. 2025, 147, 8672. 40071449 10.1021/jacs.4c18110

[28] U. Erdbrügger , T. H. Le , J. Am. Soc. Nephrol. 2016, 27, 12. 26251351 10.1681/ASN.2015010074 PMC4696584

[29] X. Zhang , Y. Jia , Z. Li , Y. Zhang , C. Wang , Y. Liang , J. Qiu , M. Sun , X. Chen , M. Huang , Y. Zhang , J. Wang , H. Liu , C. Mao , L. Han , Adv. Sci. 2025, 06167. 10.1002/advs.202506167 PMC12499472 40620113

[30] B. D. Liu , R. Akbar , A. Oliverio , K. Thapa , X. Wang , G. C. Fan , Shock 2024, 61, 175. 37878470 10.1097/SHK.0000000000002252 PMC10921997

[31] S. Li , Y. Yue , W. Wang , M. Han , X. Wan , Q. Li , X. Chen , J. Cao , Y. Zhang , J. Li , J. Li , L. Cheng , J. Yang , D. Wang , Z. Zhou , Adv. Mater. 2024, 36, 2405953. 10.1002/adma.202405953 39101293

[32] C. G. Cruz , H. M. Sodawalla , T. Mohanakumar , S. Bansal , Biology 2025, 14, 182. 40001950 10.3390/biology14020182 PMC11851951

[33] N. De Langhe , S. Van Dorpe , N. Guilbert , A. Vander Cruyssen , Q. Roux , S. Deville , S. Dedeyne , P. Tummers , H. Denys , L. Vandekerckhove , O. De Wever , A. Hendrix , Nat. Commun. 2024, 15, 9410. 39482295 10.1038/s41467-024-53279-1 PMC11528011

[34] H. J. O'Toole , N. M. Lowe , V. Arun , A. V. Kolesov , T. L. Palmieri , N. K. Tran , R. P. Carney , J. Extracell. Vesicles 2024, 13, 12506. 10.1002/jev2.12506 PMC11529045 39300768

[35] Q. Li , Z. Ou , J. Lin , D. Tang , B. He , Y. Wu , X. Huang , X. Huang , B. Ru , Q. Wang , W. Yao , B. Situ , L. Zheng , Nat. Commun. 2025, 16, 3535. 40229269 10.1038/s41467-025-58676-8 PMC11997070

[36] H. Liu , Y. Tian , C. Xue , Q. Niu , C. Chen , X. Yan , J. Extracell. Vesicles 2022, 11, 12206. 10.1002/jev2.12206 PMC8977970 35373518

[37] C. Chen , N. Cai , Q. Niu , Y. Tian , Y. Hu , X. Yan , J. Extracell. Vesicles 2023, 12, 12351. 37525378 10.1002/jev2.12351 PMC10390660

[38] Z. Ou , B. Situ , X. Huang , Y. Xue , X. He , Q. Li , D. Ou , B. He , J. Chen , Y. Huang , L. Deng , M. Zhang , Q. Wang , L. Zheng , J. Extracell. Vesicles 2023,12, 12395. 38050834 10.1002/jev2.12395 PMC10696524

[39] J. A. Welsh , G. J. A. Arkesteijn , M. Bremer , M. Cimorelli , F. Dignat‐George , B. Giebel , A. Gorgens , A. Hendrix , M. Kuiper , R. Lacroix , J. Lannigan , T. G. van Leeuwen , E. Lozano‐Andres , S. Rao , S. Robert , L. de Rond , V. A. Tang , T. Tertel , X. Yan , M. H. M. Wauben , J. P. Nolan , J. C. Jones , R. Nieuwland , E. van der Pol , J. Extracell. Vesicles 2023, 12, 12299. 10.1002/jev2.12299 PMC9911638 36759917

[40] C. Liu , N. S. Udawatte , A. Liaw , R. Staples , C. Salomon , C. J. Seneviratne , S. Ivanovski , P. Han , Adv. Healthcare Mater. 2025,14, 2403300. 10.1002/adhm.202403300 39748613

[41] G. J. Jeong , F. Khan , N. Tabassum , K. J. Cho , Y. M. Kim , Acta Biomater. 2024, 178, 13. 38417645 10.1016/j.actbio.2024.02.029

[42] C. Liu , N. Yazdani , C. S. Moran , C. Salomon , C. J. Seneviratne , S. Ivanovski , P. Han , Acta Biomater. 2024, 180, 18. 38641182 10.1016/j.actbio.2024.04.022

[43] H. B. Thapa , C. A. Passegger , D. Fleischhacker , P. Kohl , Y. C. Chen , R. Kalawong , C. Tam‐Amersdorfer , M. R. Gerstorfer , J. Strahlhofer , K. Schild‐Prüfert , E. L. Zechner , A. Blesl , L. Binder , G. A. Busslinger , L. Eberl , G. Gorkiewicz , H. Strobl , C. Högenauer , S. Schild , Nat. Commun. 2025,16, 3995. 40301356 10.1038/s41467-025-59354-5 PMC12041585

[44] M. Zu , G. Liu , H. Xu , Z. Zhu , J. Zhen , B. Li , X. Shi , M. A. Shahbazi , R. L. Reis , S. C. Kundu , G. Nie , B. Xiao , Adv. Mater. 2024,36, 2409138. 10.1002/adma.202409138 39073205

[45] N. W. Schröder , S. Morath , C. Alexander , L. Hamann , T. Hartung , U. Zähringer , U. B. Göbel , J. R. Weber , R. R. Schumann , J. Biol. Chem. 2003, 278, 15587. 12594207 10.1074/jbc.M212829200

[46] Y. Strandberg , C. Gray , T. Vuocolo , L. Donaldson , M. Broadway , R. Tellam , Cytokine 2005, 31, 72. 15882946 10.1016/j.cyto.2005.02.010

[47] P. Kumari , S. O. Vasudevan , A. J. Russo , S. S. Wright , V. Fraile‐Ágreda , D. Krajewski , E. R. Jellison , I. Rubio , M. Bauer , A. Shimoyama , K. Fukase , Y. Zhang , J. S. Pachter , S. K. Vanaja , V. A. Rathinam , Nat. Cell Biol. 2023, 25, 1860. 37973841 10.1038/s41556-023-01269-8 PMC11111309

[48] S. Gelibter , G. Marostica , A. Mandelli , S. Siciliani , P. Podini , A. Finardi , R. Furlan , J. Extracell. Vesicles 2022, 11, 12162. 10.1002/jev2.12162 PMC8804350 35102719

[49] A. Görgens , G. Corso , D. W. Hagey , R. Jawad Wiklander , M. O. Gustafsson , U. Felldin , Y. Lee , R. B. Bostancioglu , H. Sork , X. Liang , W. Zheng , D. K. Mohammad , S. I. van de Wakker , P. Vader , A. M. Zickler , D. R. Mamand , L. Ma , M. N. Holme , M. M. Stevens , O. P. B. Wiklander , J. Extracell. Vesicles 2022, 11, 12238. 10.1002/jev2.12238 PMC9206228 35716060

[50] R. Juodeikis , C. Martins , G. Saalbach , J. Richardson , T. Koev , D. J. Baker , M. Defernez , M. Warren , S. R. Carding , J. Extracell. Vesicles 2024, 13, 12406. 38240185 10.1002/jev2.12406 PMC10797578

[51] S. Davies , R. A. Marfuggi , R. A. Bright , S. Brozak , M. Osterholm , Lancet 2024, 404, 1503 39312931 10.1016/S0140-6736(24)01942-1

[52] M. W. Pletz , N. Wellinghausen , T. Welte , Intensive Care Med. 2011, 37, 1069. 21573947 10.1007/s00134-011-2245-x

[53] K. W. Witwer , E. I. Buzás , L. T. Bemis , A. Bora , C. Lässer , J. Lötvall , E. N. Nolte‐’t Hoen , M. G. Piper , S. Sivaraman , J. Skog , C. Théry , M. H. Wauben , F. Hochberg , J. Extracell. Vesicles 2013, 2. 10.3402/jev.v2i0.20360 PMC3760646 24009894

[54] S. I. Brett , F. Lucien , C. Guo , K. C. Williams , Y. Kim , P. N. Durfee , C. J. Brinker , J. I. Chin , J. Yang , H. S. Leong , Prostate 2017, 77, 1335. 28762517 10.1002/pros.23393

[55] E. I. Buzás , Z. Giricz , Front. Physiol. 2018, 9, 1479. 30405435 10.3389/fphys.2018.01479 PMC6206048

