# Supplementary material for: Dynamic Profiling of Lipoteichoic Acid (LTA) and/or Lipopolysaccharide (LPS) Positive Extracellular Vesicles in Plasma as Diagnostic and Prognostic Biomarkers for Bacterial Infection
Source: Adv Sci (Weinh). 2025 Sep 3;12(44):e06613. doi: 10.1002/advs.202506613 (PMC12667508; doi:10.1002/advs.202506613)
Supplement: Supplementary file 1 — Supporting Information [file ADVS-12-e06613-s001.docx]

**Supporting Information**


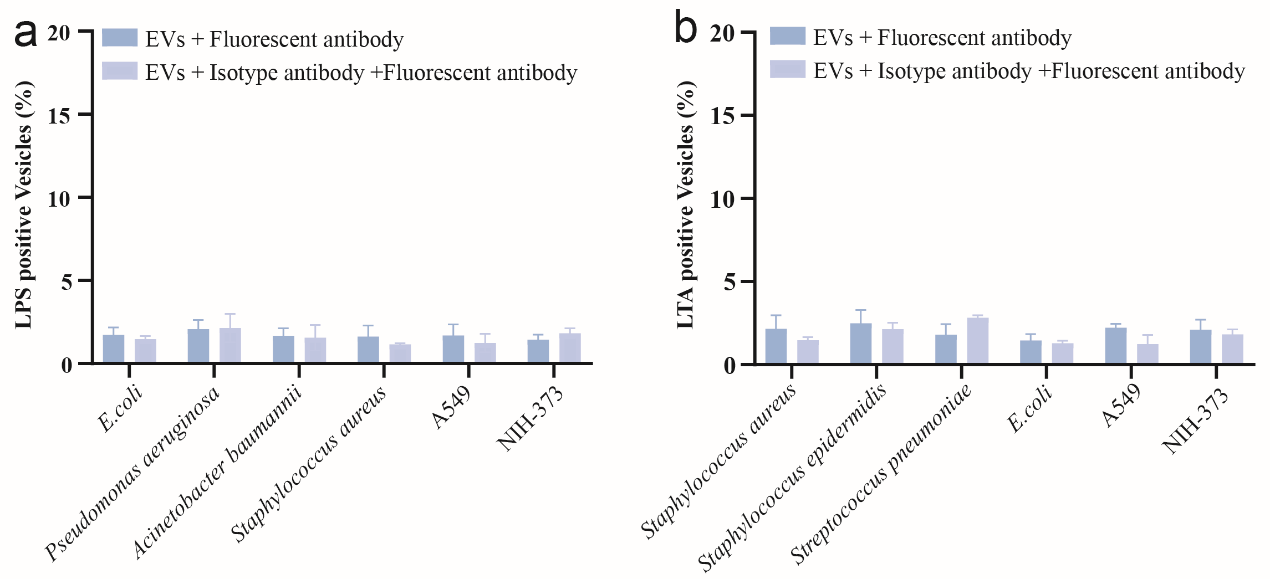


**Fig S1** **Proportions of LTA⁺ and LPS⁺ vesicles after incubation with isotype control**

(a) Proportions of LPS⁺ EVs after co-incubation with control antibodies. Data represent mean ± SD (n = 6). (b) Proportions of LTA⁺ EVs after co-incubation with control antibodies. Data represent mean ± SD (n = 6).

**
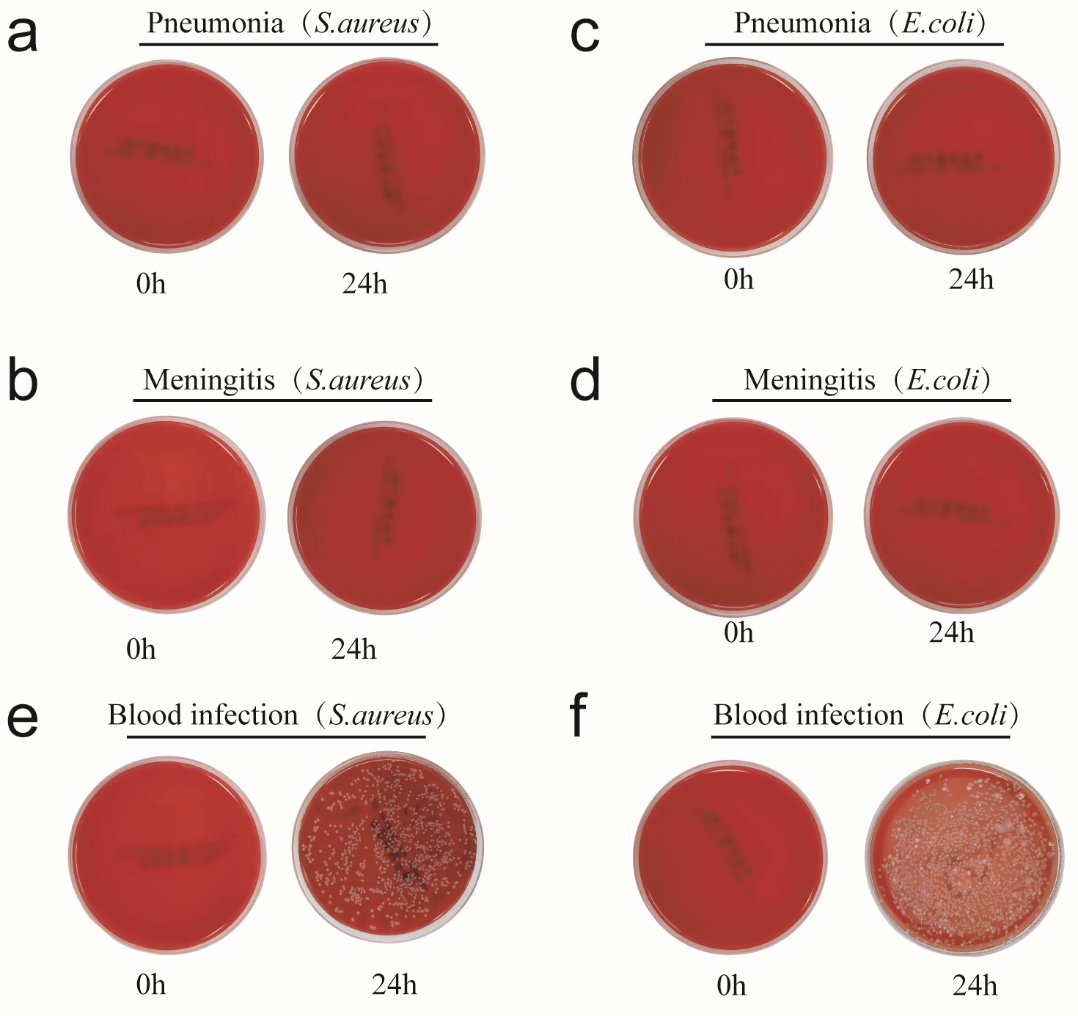
**

**Fig.S2: Blood bacterial culture results from mouse models infected with different pathogens.**

(a) Blood bacterial culture results from mice with pneumonia induced by *S. aureus*; (b) Blood bacterial culture results from mice with meningitis induced by *S. aureus*; (c) Blood bacterial culture results from mice with pneumonia induced by *E. coli*; (d) Blood bacterial culture results from mice with meningitis induced by *E. coli*; (e) Blood bacterial culture results from mice with bloodstream infection induced by *S. aureus*; (f) Blood bacterial culture results from mice with bloodstream infection induced by *E. coli*. Note: The dark shadow visible behind the blood agar plate is a watermark indicating the production date and the main components of the plate.


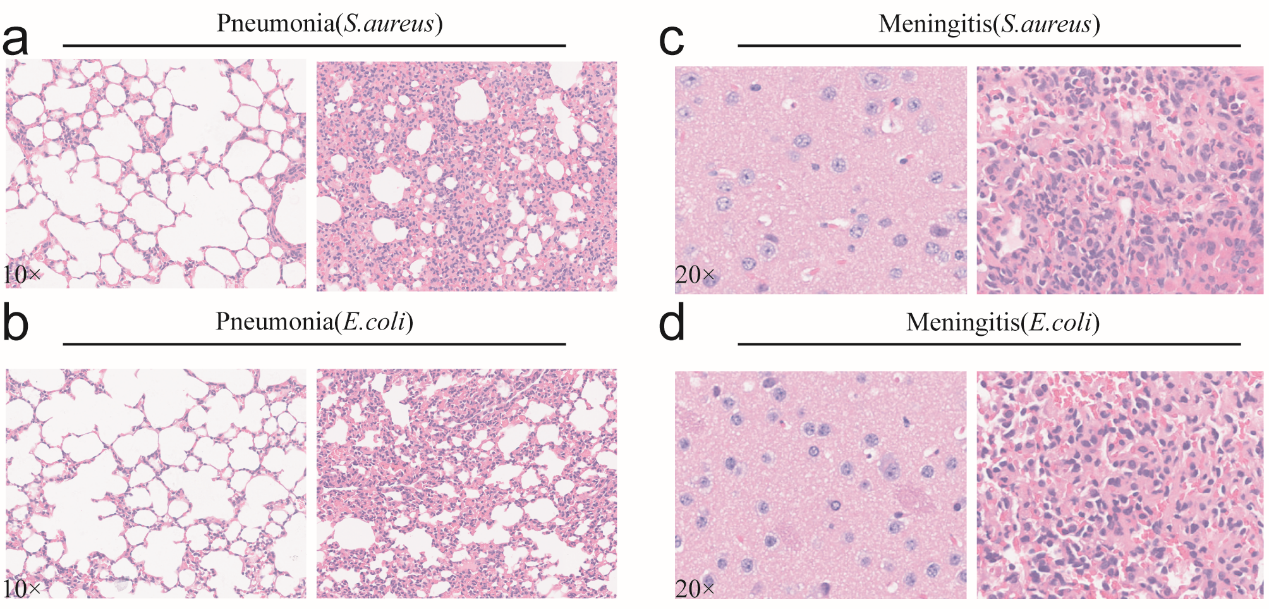


**Figure S3** Hematoxylin and Eosin (H&E) staining results of tissues from mouse models infected with different pathogens. a. H&E staining of lung tissue from mice with pneumonia induced by *S. aureus*.

b. H&E staining of lung tissue from mice with pneumonia induced by *E. coli*. c. H&E staining of brain tissue from mice with meningitis induced by *S. aureus*. d. H&E staining of brain tissue from mice with meningitis induced by *E. coli*.


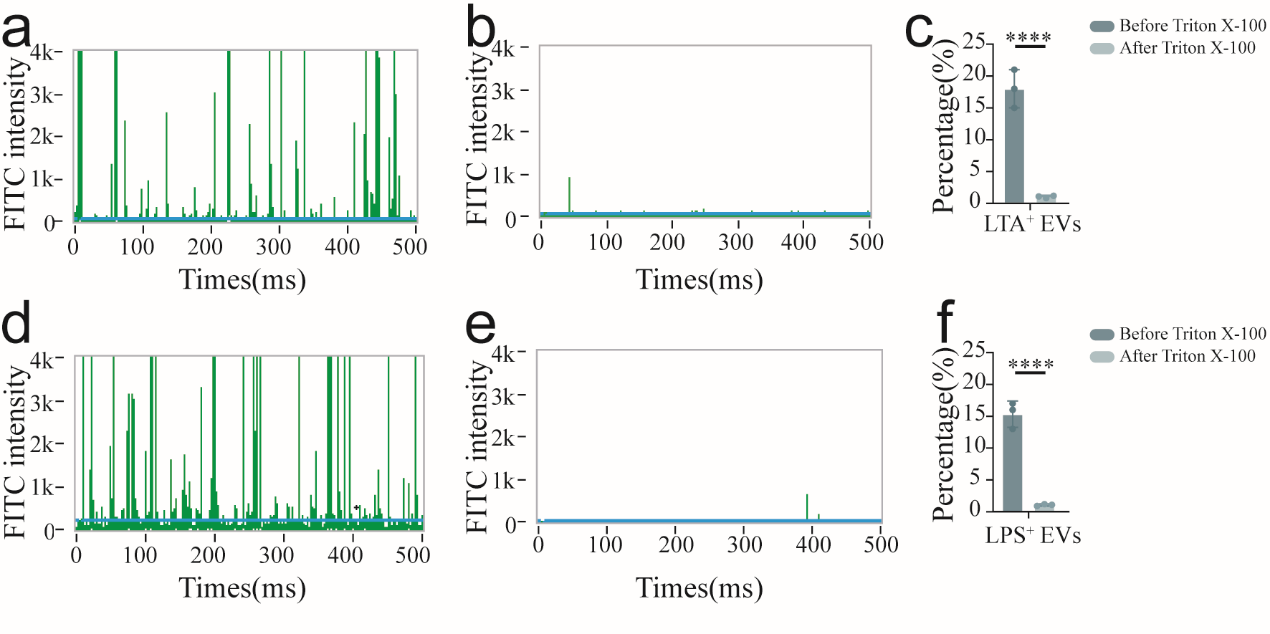


**Figure S4 Tracing LTA^+^EVs/LPS^+^EVs Before and After Triton X-100 Treatment.**

(a)Nano-FCM raw data depicting the count of LTA^+^ particles in plasma EVs before 1% Triton X-100 treatment. (b) Nano-FCM raw data depicting the count of LTA^+^ particles in plasma EVs after 1% Triton X-100 treatment. (c). Statistical graph of three independent experiments as shown in panels a and b. The statistical analysis was conducted using an independent samples t-test. (d) Nano-FCM raw data depicting the count of LPS^+^ particles in plasma EVs before 1% Triton X-100 treatment. (e) Nano-FCM raw data depicting the count of LPS^+^ particles in plasma EVs after 1% Triton X-100 treatment. (f). Statistical graph of three independent experiments as shown in panels a and b. The statistical analysis was conducted using an independent samples t-test. In all charts, n.s., *p* > 0.05; **p* < 0.05; ***p* < 0.01; ****p*＜0.001; *****p*＜0.0001.

**
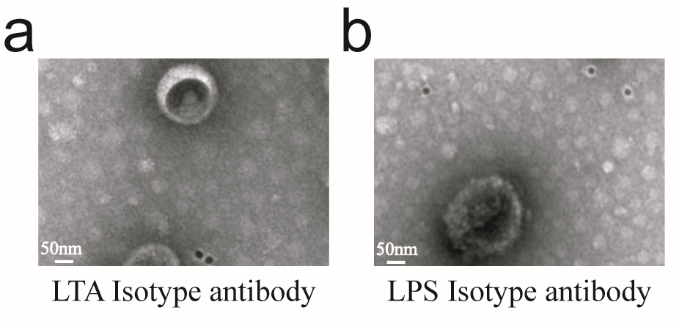
**

**Figure S5 Detection of LTA^+^ EVs in Plasma of mice co-infected with *S. aureus* and *E. coli*.** (a) Co-infected mouse plasma EVs were co-incubated with LTA isotype antibody. (b) Co-infected mouse plasma EVs were co-incubated with LPS isotype antibody.


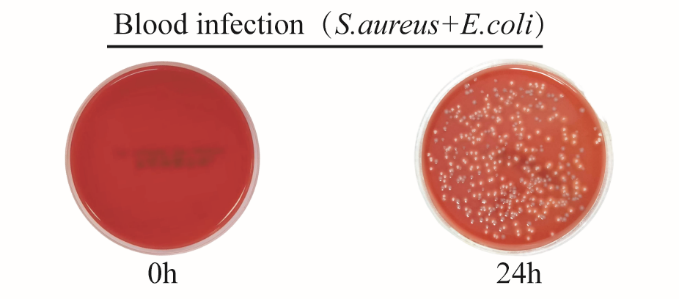


**Figure S6 Blood culture results from mice with mixed bloodstream infection by *S. aureus* and *E. coli*.** (a). Blood culture results from mice at the initial time point of infection (0 hours). (b). Blood culture results from mice 24 hours post-infection. Note: The dark shadow visible behind the blood agar plate is a watermark indicating the production date and the main components of the plate.


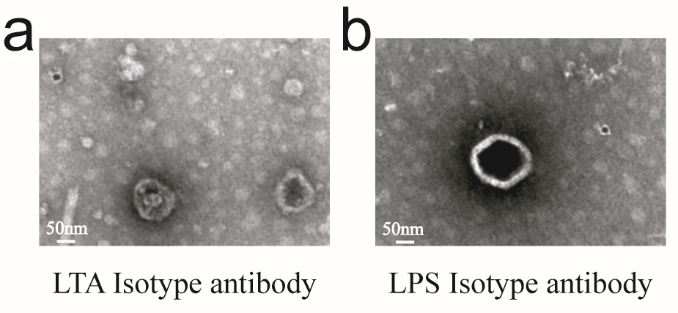


**Figure S7 Detection of LPS^+^ EVs in Plasma of mice co-infected with *S. aureus* and *E. coli*.** (a) Co-infected mouse plasma EVs were co-incubated with LTA isotype antibody. (b) Co-infected mouse plasma EVs were co-incubated with LPS isotype antibody.

**Table S1. Clinical characteristics of enrolled patients (n=78)**

| Characteristic | Value |
| --- | --- |
| Age, years | 63.63 ± 18.00 |
| Male sex, n (%) | 39 (50%) |
| WBC, ×10^9/L | 12.88 ± 6.35 |
| CRP, mg/L | 34.62 ± 31.58 |
| ICU stay, days | 2.67±4.67 |
| *S. aureus* infection, n (%) | 17, (21.79%) |
| *E. coli* infection, n (%) | 16, (20.51%) |
| Viral infection, n (%) | 15, (19.23%) |
| Crohn's disease, n (%) | 15, (19.23%) |
| Healthy, n (%) | 15 (19.23%) |
| Antibiotic use before sampling, n (%) | 18 (23.08%) |
| Survival, n (%) | 70 (89.74%) |
